# P-1030. Mortality attributable to candidemia: Results from the European Confederation of Medical Mycology Multinational Observational Cohort Study Candida III

**DOI:** 10.1093/ofid/ofae631.1220

**Published:** 2025-01-29

**Authors:** Jon Salmanton-Garcia, Oliver A Cornely, Jannik Stemler, Aleksandra Barać, Jörg Steinmann, Alena Siváková, Emin Halis Akalin, Sevtap Arıkan-Akdaglı, Laura Loughlin, Cristina Toscano, Manjusha Narayanan, Benedict Rogers, Birgit Willinger, Deniz Akyol, Emmanuel Roilides, Katrien Lagrou, Malgorzata Mikulska, Blandine Denis, Diane Ponscarme, Ulrike Scharmann, Alpay Azap, Deborah Lockhart, Tihana Bicanic, Florian Kron, Nurettin Erben, Riina Rautemaa-Richardson, Anna L Goodman, Carolina Garcia-Vidal, Cornelia Lass-Flörl, Jean-Pierre Gangneux, Lucia Taramasso, Maite Ruiz, Yael Schick, Eric Van Wijngaerden, Christopher Milacek, Daniele Roberto Giacobbe, Clare Logan, Emily Rooney, Andrea Gori, Murat Akova, Matteo Bassetti, Martin Hoenigl, Philipp Koehler

**Affiliations:** University Hospital Cologne, Cologne, Germany, Cologne, Nordrhein-Westfalen, Germany; University of Cologne, Faculty of Medicine and University Hospital Cologne, Cologne, Nordrhein-Westfalen, Germany; University Hospital Cologne, Cologne, Nordrhein-Westfalen, Germany; University Clinical Center of Serbia, Belgrade, Vojvodina, Serbia; Paracelsus Medical University, Nuremberg, Bayern, Germany; Masaryk University, Brno, Jihomoravsky kraj, Czech Republic; Bursa Uludağ University, Bursa, Bursa, Turkey; Hacettepe University, Ankara, Ankara, Turkey; Belfast Health and Social Care Trust, Belfast, Northern Ireland, United Kingdom; Centro Hospitalar de Lisboa Ocidental, Lisbon, Lisboa, Portugal; Newcastle upon Tyne Hospitals NHS Foundation Trust, Newcastle, England, United Kingdom; University Hospitals of Leicester NHS Trust, Leicester, England, United Kingdom; Medical University of Vienna, Vienna, Wien, Austria; Doctor, İzmir, Izmir, Turkey; Hippokration General Hospital, Thessalonike, Thessaloniki, Greece; University Hospitals Leuven & KU Leuven, Leuven, Vlaams-Brabant, Belgium; University of Genoa, IRCCS San Martino Hospital, Genoa, Liguria, Italy; Hôpital Saint-Louis, Paris, Ile-de-France, France; Hôpital Saint-Louis, Paris, Ile-de-France, France; University of Duisburg-Essen, Essen, Nordrhein-Westfalen, Germany; Ankara University, Ankara, Ankara, Turkey; University of Aberdeen, Aberdeen, Scotland, United Kingdom; St Georges University Hospital, London, United Kingdom, London, England, United Kingdom; VITIS Healthcare Group, Cologne, Nordrhein-Westfalen, Germany; Eskisehir Osmangazi University, Eskisehir, Eskisehir, Turkey; University of Manchester, Manchester, England, United Kingdom; Guy's and St Thomas' NHS Foundation Trust and University College, London, London, England, United Kingdom; Hospital Clínic de Barcelona, Barcelona, Catalonia, Spain; Medical University of Innsbruck, Innsbruck, Tirol, Austria; University of Rennes, Rennes, Bretagne, France; Fondazione IRCCS Ca' Granda-Ospedale Maggiore Policlinico di Milano, Milan, Lombardia, Italy; University Hospital Virgen del Rocio, Seville, Andalucia, Spain; University of Cologne, Cologne, Nordrhein-Westfalen, Germany; KU Leuven, Leuven, Vlaams-Brabant, Belgium; Medical University of Vienna, Vienna, Wien, Austria; University of Genoa, Genoa, Liguria, Italy; St. George’s University Hospital National Health Service (NHS) Foundation Trust, London, England, United Kingdom; Manchester University NHS Foundation Trust, Manchester, England, United Kingdom; Infectious Diseases and Immunopathology, Department of Clinical Sciences, Università di Milano, Luigi Sacco Hospital, Milan, Italy, Milano, Lombardia, Italy; Hacettepe University, Ankara, Ankara, Turkey; Department of Health Science (DISSAL), Infectious Diseases Unit,, Genova, Liguria, Italy; Medical University of Graz, Department of Internal Medicine, Division of Infectious Diseases, Graz, Steiermark, Austria; University Hospital Cologne, Cologne, Germany, Cologne, Nordrhein-Westfalen, Germany

## Abstract

**Background:**

Despite advances in antifungals, Candida infections still have a high mortality rate of up to 40%. The ECMM Candida III study in Europe investigated the changing epidemiology and outcomes, highlighting the need to understand and manage these infections.

**Methods:**

In this observational cohort study, participating hospitals enrolled the first ten consecutive adults with confirmed candidemia. Data collected included patient demographics, risk factors, hospital stay length (with a 90-day follow-up), diagnostic procedures, Candida species, treatment details, and outcome. Controls were matched in a 1:1 ratio from the same hospitals, ensuring similarity in age, underlying illness, ICU versus normal ward stay, and recent major surgery. The study described overall and attributable mortality and assessed survival probability for both cases and controls.

**Results:**

The study included 171 pairs consisting of patients with candidemia and matched controls from 28 institutions. In those with candidemia, overall mortality was 40.4%. The attributable mortality was 18.1% overall but differed among the causative Candida species (7.7% for Candida albicans, 23.7% for Candida glabrata, 7.7% for Candida parapsilosis, and 63.6% for Candida tropicalis). Regarding risk factors, the presence of central venous catheter, total parenteral nutrition, and acute or chronic kidney disease were significantly more common in cases versus controls. Length of hospitalization and ICU stay were significantly longer in candidemia cases (20 days (IQR 10-33) vs. 15 days (IQR 7-28); p=0.004).

**Conclusion:**

Although overall mortality remains high in this matched case/control analysis, attributable mortality has decreased compared to historical cohorts. This may be due to a better prognosis for candidemia caused by Candida albicans, which has an attributable mortality of 7.7%, while candidemia cases caused by non-albicans Candida exhibit higher attributable mortality.

**Disclosures:**

**Oliver A. Cornely, Prof. Dr.**, Abbott: Honoraria|Abbvie: Advisor/Consultant|Abbvie: Honoraria|AiCuris: Advisor/Consultant|Akademie fur Infektionmedizin: Honoraria|Al-Jazeera Pharmaceuticals/Hikma: Honoraria|amedes: Honoraria|AstraZeneca: Honoraria|Basilea: Advisor/Consultant|Biocon: Advisor/Consultant|BMBF: Grant/Research Support|Boston Strategic Partners: Advisor/Consultant|CIdara: Advisor/Consultant|CIdara: Expert Testimony|CIdara: Grant/Research Support|CIdara: Participation on a DRC or DSMB|CoRe Consulting: Stocks/Bonds (Private Company)|Deutscher Arzteverlag: Honoraria|DZIF: Grant/Research Support|EasyRadiology: Stocks/Bonds (Private Company)|EU-DG RTD: Grant/Research Support|F2G: Grant/Research Support|Gilead: Advisor/Consultant|Gilead: Grant/Research Support|Gilead: Honoraria|Grupo Biotoscana/United Medical/Knight: Honoraria|GSK: Advisor/Consultant|GSK: Honoraria|IQVIA: Advisor/Consultant|IQVIA: Participation on a DRC or DSMB|Janssen: Advisor/Consultant|Janssen: Participation on a DRC or DSMB|Matinas: Advisor/Consultant|MedPace: Advisor/Consultant|MedPace: Grant/Research Support|MedPace: Participation on a DRC or DSMB|Medscape/WebMD: Honoraria|MedUpdate: Honoraria|Menarini: Advisor/Consultant|Moderna: Honoraria|Molecular Partners: Advisor/Consultant|MSD: Grant/Research Support|MSD: Honoraria|MSG-ERC: Advisor/Consultant|Mundipharma: Advisor/Consultant|Mundipharma: Grant/Research Support|Mundipharma: Honoraria|Noscendo: Honoraria|Noxxon: Advisor/Consultant|Octapharma: Advisor/Consultant|Octapharma: Grant/Research Support|Pardes: Advisor/Consultant|Partner Therapeutics: Advisor/Consultant|Patent: US18/562644|Paul-Martini-Stiftung: Honoraria|Pfizer: Advisor/Consultant|Pfizer: Grant/Research Support|Pfizer: Honoraria|PSI: Advisor/Consultant|PSI: Participation on a DRC or DSMB|Pulmocide: Participation on a DRC or DSMB|Sandoz: Honoraria|Scynexis: Advisor/Consultant|Scynexis: Grant/Research Support|Seqirus: Advisor/Consultant|Seqirus: Honoraria|Seres: Advisor/Consultant|Shionogi: Advisor/Consultant|Shionogi: Honoraria|streamedup!: Honoraria|The Prime Meridian Group: Advisor/Consultant|Touch Independent: Honoraria|Vitis: Honoraria **Jean-Pierre Gangneux, Prof.**, Gilead: Honoraria|MundiPharma: Advisor/Consultant|MundiPharma: Honoraria|Pfizer: Honoraria|Shionogi: Honoraria **Matteo Bassetti, PhD**, Angelini: Advisor/Consultant|Angelini: Honoraria|Astellas: Advisor/Consultant|Astellas: Honoraria|bioMerieux: Advisor/Consultant|bioMerieux: Honoraria|Cidara: Advisor/Consultant|Cidara: Honoraria|Gilead: Advisor/Consultant|Gilead: Honoraria|Menarini: Advisor/Consultant|Menarini: Honoraria|MSD: Advisor/Consultant|MSD: Honoraria|Nabriva: Advisor/Consultant|Nabriva: Honoraria|Pfizer: Advisor/Consultant|Pfizer: Honoraria|Tetraphase: Advisor/Consultant|Tetraphase: Honoraria

